# Dynamic Proteomics of Herpes Simplex Virus Infection

**DOI:** 10.1128/mBio.01612-17

**Published:** 2017-11-07

**Authors:** Nir Drayman, Omer Karin, Avi Mayo, Tamar Danon, Lev Shapira, Dor Rafael, Anat Zimmer, Anat Bren, Oren Kobiler, Uri Alon

**Affiliations:** a Department of Molecular Cell Biology, Weizmann Institute of Science, Rehovot, Israel; b Department of Clinical Microbiology and Immunology, Sackler School of Medicine, Tel Aviv University, Tel Aviv, Israel; New York University School of Medicine; Columbia University

**Keywords:** cell cycle, geminin, RFX7, RPAP3, SLTM, single-cell infection, systems biology, YTHDC1, cell-to-cell variability, herpes simplex virus, mitosis

## Abstract

The cellular response to viral infection is usually studied at the level of cell populations. Currently, it remains an open question whether and to what extent cell-to-cell variability impacts the course of infection. Here we address this by dynamic proteomics—imaging and tracking 400 yellow fluorescent protein (YFP)-tagged host proteins in individual cells infected by herpes simplex virus 1. By quantifying time-lapse fluorescence imaging, we analyze how cell-to-cell variability impacts gene expression from the viral genome. We identify two proteins, RFX7 and geminin, whose levels at the time of infection correlate with successful initiation of gene expression. These proteins are cell cycle markers, and we find that the position in the cell cycle at the time of infection (along with the cell motility and local cell density) can reasonably predict in which individual cells gene expression from the viral genome will commence. We find that the onset of cell division dramatically impacts the progress of infection, with 70% of dividing cells showing no additional gene expression after mitosis. Last, we identify four host proteins that are specifically modulated in infected cells, of which only one has been previously recognized. SUMO2 and RPAP3 levels are rapidly reduced, while SLTM and YTHDC1 are redistributed to form nuclear foci. These modulations are dependent on the expression of ICP0, as shown by infection with two mutant viruses that lack ICP0. Taken together, our results provide experimental validation for the long-held notion that the success of infection is dependent on the state of the host cell at the time of infection.

## IMPORTANCE

High-throughput assays have revolutionized many fields in biology, both by allowing a more global understanding of biological processes and by deciphering rare events in subpopulations. Here we use such an assay, dynamic proteomics, to study viral infection at the single-cell level. We follow tens of thousands of individual cells infected by herpes simplex virus using fluorescence live imaging. Our results link the state of a cell at the time of virus infection with its probability to successfully initiate gene expression from the viral genome. Further, we identified three cellular proteins that were previously unknown to respond to viral infection. We conclude that dynamic proteomics provides a powerful tool to study single-cell differences during viral infection.

## INTRODUCTION

Viral infection is a heterogeneous process. For example, the number of viral progeny produced by individual cells spans several orders of magnitude as first described for bacteriophages in the 1940s (1) and more recently for some RNA mammalian viruses (2–6).

Variability also exists in the outcome of infection, as some cells in the population become successfully infected, while others resist the infection. A well-known source of this variability is stochastic, emanating from the random distribution of the number of viruses that individual cells encounter (7–9). Another source is the heterogeneity in the virus population, with some virus particles being unable or less fit to establish infection (10–12). Currently, it remains unclear whether and to what extent other factors help to shape the outcome of infection.

One possible determinant might be the state of the host cell at the time of infection. At the single-cell level, genetically identical cells can show a high degree of cell-to-cell variability, which arises from the continuous progress through the cell cycle (in actively dividing cells) and from stochastic changes in mRNA transcription and protein translation. The effect of such cell-to-cell variability has been studied in several systems and was shown to influence many biological processes (13–17).

Supporting a role for the state of the host cell in shaping the outcome of infection, Snijder et al. found that cellular features such as the cell size and shape, lipid uptake, and location in the colony differ between successfully infected and noninfected cells (18, 19). However, their analysis relied on cells observed at the endpoint of the infection process, making it hard to deduce whether the observed differences between the cells are the cause or consequence of a successful infection.

There is a lack of experiments that directly address the question of how the host cell state affects infection outcome. Answering this requires a system that continuously follows individual cells during viral infection.

Here we aim to study how the state of the host cell shapes gene expression from the viral genome, using herpes simplex virus 1 (HSV-1) as a model system. HSV-1 is a common human pathogen that belongs to the *Herpesviridae* family and serves as the prototypic virus for studying alphaherpesvirus infection. HSV-1 *de novo* infection has a lytic phase and a latent phase. In the lytic phase, the virus infects epithelial cells at the site of contact, where it replicates, destroys the host cell, and releases viral progeny. The latent phase is restricted to the host neurons, in which the virus remains silent throughout the host’s life, with occasional reactivation. Here we study only the lytic part of the virus life cycle. In the majority of cases, the initial site of HSV-1 infection is the vermillion border of the lip (20), where the virus can infect both fibroblasts and keratinocytes (21, 22).

HSV-1 infection is known to interfere with the natural progress of the cell cycle, with infection usually resulting in cell cycle arrest at the G_1_/S (23–26) or G_2_/M (26) checkpoints. The major protein responsible for the dysregulation of the cell cycle is the immediate early protein ICP0 (26–28), although other viral proteins have also been implicated (29, 30).

To initiate a cellular infection, the virus has to bind to its receptors, enter the cytoplasm, travel to the nuclear pore, inject its linear double-stranded DNA into the host nucleus, and initiate viral gene expression (31). Once inside the nucleus, the naked viral DNA associates with host histones to form nucleosomes (32, 33). The probability of initiating immediate early gene expression depends on interactions between the viral DNA, the tegument protein VP16, and host factors (34). Viral immediate early proteins activate expression of the viral early and late genes in addition to shutting down host defense mechanisms. Both intrinsic and innate immunity are inhibited by the viral immediate early protein ICP0 (35). A detailed proteomic study of HSV-1 infection at the population level has recently found that 6.6% of the host proteins studied (286 out of 4,326) reacted to infection (36). Thus, like all viruses, HSV-1 closely interacts with its host cell’s machinery, and specific mechanisms in the host cell are likely to modify the outcome of the infection.

**FIG 1:**
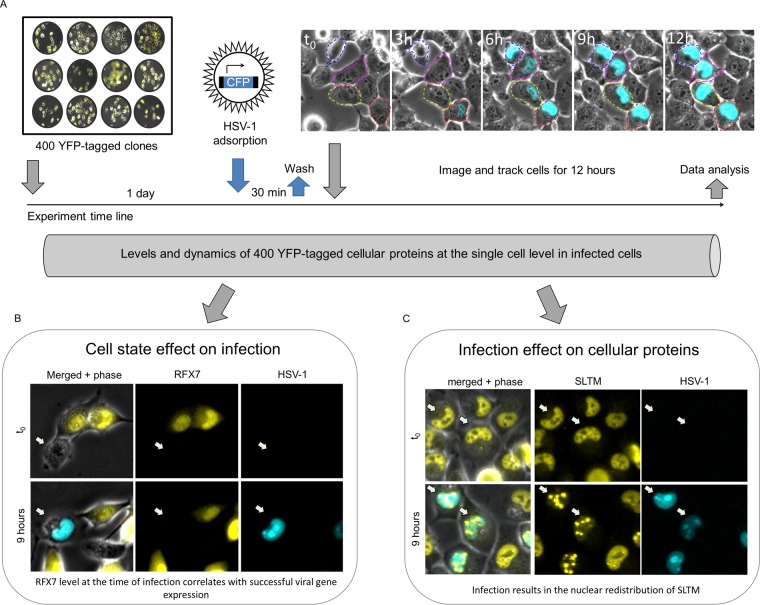
Dynamic proteomics to study virus-host interactions in single cells. (A) Schematic representation of the screen. A CFP-expressing HSV-1 was allowed to adsorb to clones seeded in 12-well plates for 30 min and washed out, and then the cells were imaged every 20 min for 12 h. Overall, more than 50,000 single cells from 400 different YFP-expressing clones were monitored. (B) An example of a cellular protein whose level correlates with successful initiation of gene expression from the viral genome. RFX7 (yellow) levels are highly heterogeneous in the cell population. Cells with low levels of RFX7 at the time of infection are more likely to express CFP. See Fig. 2 for more information. (C) An example of a cellular protein affected by HSV-1 infection. The nuclear localization of SLTM (yellow) changes upon infection by the virus. See Fig. 7 for more information.

Here we employ dynamic proteomics (14, 37, 38) to monitor 400 yellow fluorescent protein (YFP)-tagged cellular proteins in individual cells infected by HSV-1 (Fig. 1A). We aim to identify proteins and other cellular parameters (such as shape and movement) whose cell-to-cell variability at the time of infection correlates with successful initiation of gene expression from the viral genome (Fig. 1B). While not our primary focus, the screen also identifies proteins whose levels or localization are actively modulated by the virus (Fig. 1C).

We find that the concentrations of two cell cycle-regulated proteins at the time of infection (RFX7 and geminin) are predictive of successful gene expression by HSV-1. The position in the cell cycle at the time of infection along with two other factors (local cell density and cell motility) can correctly predict whether a cell will initiate gene expression from the viral genome in about 60% of the cells, showing that the probability of successful infection is not identical among single cells. We additionally identify three previously unknown cellular proteins that respond to HSV-1 infection (RPAP3, SLTM, and YTHDC1) and show that their modulation depends on the expression of the viral E3 ubiquitin-ligase ICP0.

## RESULTS

### Dynamic proteomics of human cells following HSV-1 infection.

To experimentally assess the impact of the cellular state at the time of virus encounter on gene expression from the HSV-1 genome, we employed the dynamic proteomic approach (Fig. 1). This approach relies on a library of annotated clones and automated image analysis to monitor protein dynamics and cell behavior at the level of individual cells over time (39). The clones in the library were derived from the H1299 human cell line and express two mCherry-tagged proteins (one bright in the nucleus and the other dim in the cytoplasm) that are used for automated segmentation and tracking of the cells. Each clone additionally expresses a unique YFP-tagged protein from its endogenous chromosomal location, such that the tagged protein retains its native transcriptional control.

We performed a primary screen, in which we infected 400 clones from the library with a cyan fluorescent protein (CFP)-expressing HSV-1 at a low multiplicity of infection (MOI) of 0.5, imaging the cells every 20 min for 12 h (Fig. 1A). Throughout this work, we use CFP (expressed from the viral genome) as a surrogate marker for viral gene expression. We determined whether gene expression from the viral genome was successfully initiated based on the cell’s CFP levels 9 h after adsorption. The timing and level of CFP expression are similar to those of the viral immediate early protein ICP4 (see Fig. S1 in the supplemental material), the main HSV-1 transactivator (40). The percentage of CFP-positive (CFP^+^) cells varied among different clones with a mean of 35% ± 13%. This design allowed us to compare CFP^+^ and CFP-negative (CFP^−^) cells side by side, while monitoring infection progress in real time. Note that the particle-to-infectious unit ratio of HSV-1 is estimated to be 50 to 200, suggesting that all the cells encountered numerous viral particles. H1299 cells are fully permissive for HSV-1 infection, and new viral progeny can be detected in infected cells from 6 h postinfection (Fig. S2).

10.1128/mBio.01612-17.1 FIG S1  CFP expression correlates with the expression of the HSV-1 immediate early protein ICP4. (A) Cells infected with HSV-1 expressing CFP were fixed, stained for ICP4, and imaged 2 to 4 h after adsorption, showing that CFP expression (shown in green for easier visualization) is similar to that of ICP4. (B and C) Quantification of the levels of ICP4 and CFP from 60 cells at the 4-h time point. The graph in panel B is shown on a log-log scale, and the graph in panel C shows the boxed area on a linear-linear scale. Correlation was calculated on the raw data (not log transformed) and is significant (Pearson coefficient of 0.86 and *P* value of 7 × 10^−19^). The average fold difference in expression levels is 1.4 ± 0.5 (mean ± standard deviation [SD]). Download FIG S1, TIF file, 0.2 MB. Copyright © 2017 Drayman et al. 2017 Drayman et al. This content is distributed under the terms of the Creative Commons Attribution 4.0 International license.

10.1128/mBio.01612-17.2 FIG S2  New viral progeny are seen from 6 h postinfection. H1299 cells were infected with CFP expressing HSV-1 at an MOI of 1. At different time points postinfection, progeny viruses were collected and titrated on Vero cells. An average of three technical replicates per time point is shown. From 4 to 6 h postinfection, there is an approximately 1-log-unit increase in infectivity, showing that new viral progeny have been produced. Download FIG S2, TIF file, 0.2 MB. Copyright © 2017 Drayman et al. 2017 Drayman et al. This content is distributed under the terms of the Creative Commons Attribution 4.0 International license.

Using custom software, we segmented and tracked ~52,000 cells. For each cell at each time point, we recorded its protein levels (YFP for the host protein and CFP for the viral marker) and other features such as the cell’s position, shape, and size. We calculated the effect size of each protein on successful initiation of gene expression by looking at the difference in YFP concentration at time zero between cells that will become CFP^+^ or CFP^−^. In other words, the effect size is the difference in the mean protein concentration between CFP^+^ and CFP^−^ cells at the first time point of the experiment, which is the end of the virus adsorption period.

**FIG 2:**
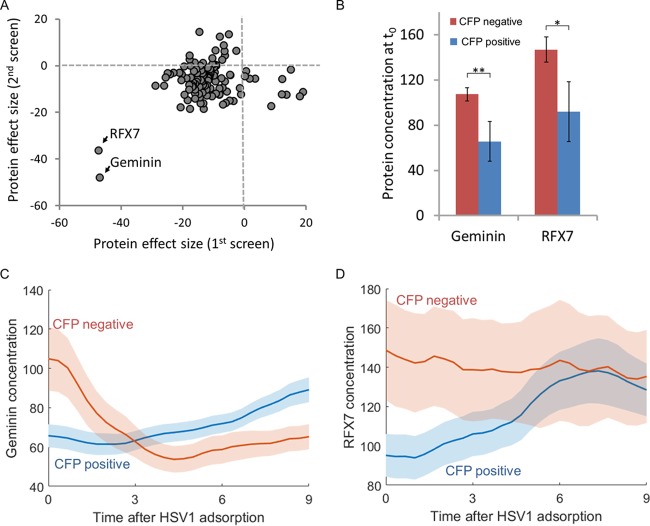
RFX7 and geminin concentrations at the time of infection correlate with successful initiation of gene expression from the viral genome. (A) The protein effect size (percent difference between CFP^+^ and CFP^−^ cells at time zero) of 115 clones in the primary screen and an independent validation screen identified two proteins (RFX7 and geminin) that correlate with successful gene expression from the HSV-1 genome. (B) Geminin and RFX7 concentration (mean ± standard error of the mean [SEM] [error bar]) in CFP^+^ cells (blue bars) or CFP^−^ cells (red bars) at time zero. Values that are significantly different by one-tailed *t* test are shown by a bar and asterisk as follows: *, *P* < 0.05; **, *P* < 0.01. (C and D) Geminin (C) and RFX7 (D) concentration (mean ± SEM) over time in CFP^+^ cells (blue lines) or CFP^−^ cells (red lines). The mean values are indicated by the colored lines, and the SEMs are indicated by the colored areas.

From the 400 proteins in the initial screen, we chose 115 for independent validation in a secondary screen. We included the top hits from the primary screen, as well as some randomly chosen proteins. The combined results of the screens are presented in Fig. 2A. The majority of proteins showed a repeatable small negative effect of size (less than 20% difference in YFP concentration between CFP^+^ and CFP^−^ cells at time zero). Two proteins were clear outliers; both RFX7 and geminin were much less abundant (37 to 47% lower) in cells that will become CFP^+^ (Fig. 2A and B). This difference in protein concentration was most notable at the time of infection and lasted for about 3 h into the infection process (Fig. 2C and D). Thus, we identified two proteins whose concentration at the time of infection is correlated with the probability of a cell to successfully initiate gene expression from the HSV-1 genome.

### The position in the cell cycle affects the probability to successfully initiate gene expression from the viral genome.

**FIG 3:**
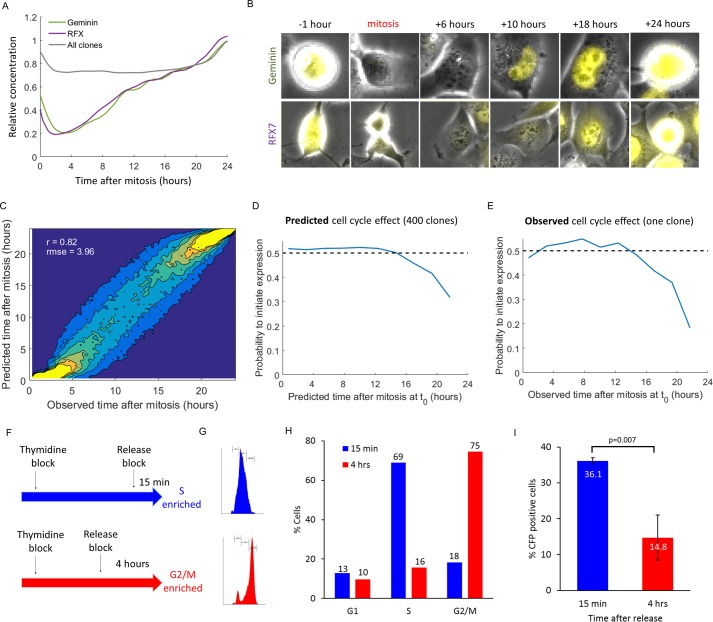
The cell cycle stage at the time of infection affects the probability of initiation of gene expression from the viral genome. (A) Geminin and RFX7 concentrations are dependent on the cell cycle. The gray line depicts the median behavior of all other clones in the screen. (B) Representative images of cells expressing YFP-tagged geminin (YFP-geminin) (top row) or YFP-RFX7 (bottom row) at different times after mitosis. (C) Cell cycle prediction from still images. A data set of noninfected cells that divided during the time-lapse movies was used to train a random forest predictor. It is composed of 500 decision trees, based on 30 image analysis features. Panel C shows the performance of the predictor on an independent test set. The predictor calculates the time from last mitosis with a root mean square error (rmse) of 3.96. The Pearson correlation coefficient is 0.82. (D) The probability of initiating CFP expression as a function of the predicted time from mitosis, calculated for all the cells in our screen based on the cell cycle predictor shown in panel C. The dashed black line denotes an infection probability of 0.5 (*P* = 3 × 10^−25^ by the Kolmogorov-Smirnov test). (E) The probability of initiating CFP expression as a function of the time from mitosis, extracted from long time-lapse movies in which the cells were imaged for 24 h before virus infection (*P* = 0.04 by the Kolmogorov-Smirnov test). (F) Schematic representation of enriching cells for the S or G_2_/M phases using a thymidine block. (G) Cell cycle analysis of synchronized cells, 15 min (top) or 4 h (bottom) after release from thymidine block. (H) Proportions of cells in the G_1_, S, and G_2_/M phases 15 min or 4 h after release from the thymidine block. (I) Percentage of CFP-positive cells (mean ± SEM from four biological repeats) 9 h after infection of cells 15 min or 4  after release from the thymidine block. The values were significantly different (*P* = 0.007) by a two-sample *t* test.

Geminin is a substrate of the anaphase-promoting complex (41), and as such, its level correlates with the cell cycle. RFX7 is a member of a transcription factor family that binds the X-box motif, which is important for the regulation of immune genes such as major histocompatibility complex (MHC) class II (42). Geminin and RFX7 levels show a similar cell cycle-dependent profile (Fig. 3A). Both are rapidly degraded after mitosis and accumulate toward the next mitosis (Fig. 3B). The facts that the concentrations of geminin and RFX7 are lower in cells that will become CFP^+^ and that their levels are lowest after mitosis suggest that as the cells progress through the cell cycle, they become less likely to successfully initiate gene expression from the viral genome.

Next, we identified the position of each cell in the cell cycle in our screen at the time of infection. To do so, we relied on a supervised machine learning approach to infer the position of a cell in the cell cycle from its still image, using various cellular features such as the nuclear size and cell roundness, similar to approaches taken by others (43–45). We trained and tested the predictor performance on a subset of cells that divided during our time-lapse imaging, so that we could compare the prediction with the actual time after mitosis. The predictor correctly assigns the time from the last mitosis as seen in Fig. 3C (Pearson correlation coefficient of 0.82). Using this approach to assign a cell cycle time for each cell in our data set, we measured the probability of initiating gene expression from the viral genome as a function of the predicted time from the last mitosis (Fig. 3D). In agreement with the lower concentrations of RFX7 and geminin, the probability is around 0.5 for the first 12 h after mitosis and then begins a steady decline, reaching a minimum of 0.32 toward the next mitosis.

To ascertain the results obtained from the predicted cell cycle times, we performed a long time-lapse imaging experiment, in which the cells were imaged for 24 h prior to infection, infected, and continued to be imaged for an additional 12 h. This allowed us to determine the time from the last mitosis of each cell at the time of infection directly (rather than predicting it from the cell image). We measured the probability of initiating gene expression from the viral genome as a function of the observed time from the last mitosis (Fig. 3E), which was highly similar to the results obtained from the predicted values (Fig. 3D), the only difference being that the probability toward the next mitosis is lower (0.18 compared to 0.32).

We additionally validated this cell cycle effect by an orthogonal approach, using the thymidine block protocol to synchronize cells to the G_1_/S checkpoint (46). We infected cells either 15 min or 4 h after release from the thymidine block, enriching cells for the S or G_2_/M phases, respectively (Fig. 3F to H). We quantified the percentage of CFP-positive cells at 9 h postinfection and found that S-phase-enriched cells had 2.4-fold more CFP-positive cells than G_2_/M-enriched cells (Fig. 3I, 36.1% versus 14.8% [*P* value of 0.007]).

Taken together, our results demonstrate that the cell cycle position at the time of infection influences the probability of gene expression from the HSV-1 genome.

### Gene expression from the HSV-1 genome also depends on the cell motility and local cell density.

In addition to the information about specific protein levels, we also measured multiple cellular parameters from the image of each cell. These parameters include features of cell morphology (such as cell size and roundness), texture (such as homogeneity and contrast), motility (cell velocity), and the local cell density (number of neighboring cells).

**FIG 4:**
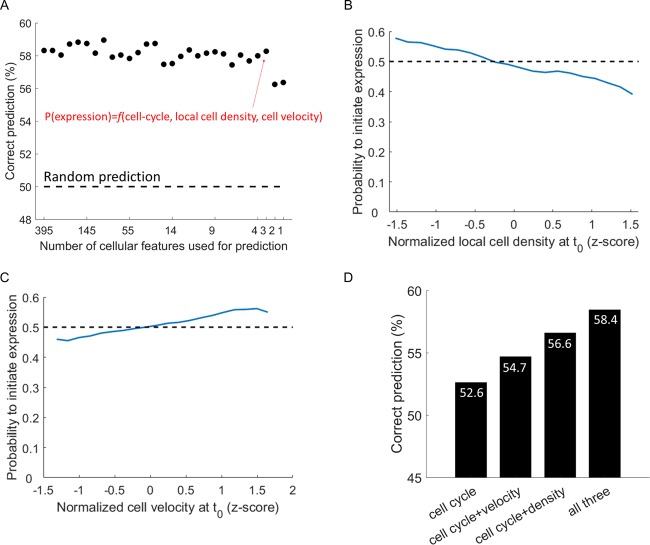
Gene expression success can be predicted to a degree based on three factors. (A) The correct prediction rate of a machine learning algorithm as a function of the number of features used in the prediction. The thin red arrow indicates the model with minimal number of features that still performs well. The model computes the probability of a single cell successfully initiating gene expression from the HSV-1 genome, P(expression), as a function (*f*) of three cellular parameters: cell cycle, local cell density, and cell velocity. (B and C) The probability of initiating CFP expression as a function of local cell density (B) or velocity (C) at the time of infection. The dashed black lines denote an infection probability of 0.5. (D) The correct classification rate increases when adding the cell velocity and local cell density data to the cell cycle data.

To test whether any of these features influence gene expression from the viral genome, we again employed a supervised machine learning approach. We aggregated all cells into a single data set (as these features are common to all clones, not depending on the YFP-tagged proteins) and divided it into independent training and test sets. We trained a logistic regression algorithm on the training set and tested its performance on the test set. We sequentially removed the least informative features until a minimal set of features that can predict gene expression outcome remained. We found that three factors—cell cycle position, cell velocity, and local cell density—carry most of the information (Fig. 4A). In other words, knowing the values of these three factors at the time of infection is sufficient to correctly predict which cells will successfully initiate gene expression (the correct classification rate is 59%, significantly better than random [*P* = 2.8 × 10^−66^ by a chi-square test]).

Local cell density (the number of neighboring cells) is negatively correlated with CFP expression probability (Fig. 4B), and the cells with the fewest neighbors were 50% more likely to successfully express CFP than cells with the most neighbors (infection probability of 0.6 and 0.4, respectively). Using only the local cell density to predict expression outcome results in 56% correct classification (*P* = 2 × 10^−37^).

Cell velocity is positively correlated with CFP expression probability (Fig. 4C), with the fastest moving cells being 30% more likely to successfully express CFP than the slowest cells (0.57 and 0.43, respectively). Using only the cell velocity to predict expression outcome results in 55% correct classification (*P* = 1 × 10^−23^).

Both the cell velocity and local cell density contribute to the cell’s probability to initiate gene expression independently from each other and from the cell cycle, as evident by the increase in the correct prediction rate upon their addition to the model (Fig. 4D). This partial success of machine learning in predicting which cells will initiate gene expression from the HSV-1 genome suggests that infection outcome is not random and that the cell state at the time of infection is an important determinant of infection success.

### Cell division after initiation of gene expression from the viral genome results in attenuation of expression kinetics.

After establishing that cell-to-cell variability affects the success of gene expression from the HSV-1 genome and that the cell cycle is an important determinant of gene expression, we turn our attention to study the kinetics of gene expression. To do so, we took advantage of the continuous monitoring of the cells in our screen, which enabled us to explore the kinetics of gene expression (in the fraction of cells that successfully initiated viral gene expression), using CFP expression as a surrogate marker (Fig. S1) (9).

**FIG 5:**
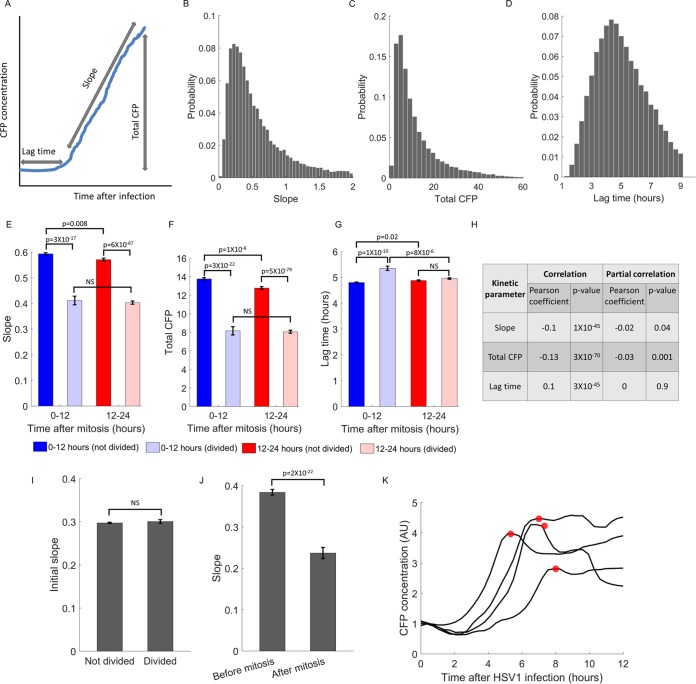
Cell division decreases expression kinetics. (A) A schematic representation of a typical trajectory of CFP concentration after infection. The trajectory can be described by three kinetic parameters: (i) the lag time between virus adsorption and the start of CFP accumulation, (ii) the rate of CFP accumulation (the slope of the line), and (iii) the total CFP accumulated in the cells during the 12 h of imaging. (B to D) Histograms showing the distribution of the kinetic parameters among individual cells. The plots are shown for slope (B), total CFP (C), and lag time (D). (E to G) We divided the cells in each cell cycle group (early [0 to 12 h after mitosis] and late [12 to 24 h after mitosis]) according to whether or not they divided after infection (2% for the early cell cycle, 12% for the late) and compared the three kinetic parameters of gene expression: slope (E), total CFP (F), and lag time (G). The different colored bars indicate the time and division status of the cells as follows: dark blue bars, early, not divided; light blue bars, early, divided; dark red bars, late, not divided; pink bars, late, divided. The mean ± SEM are shown for each group, and the *P* values were calculated by two-sample *t* tests and Bonferroni corrected for multiple hypotheses testing. NS, not significantly different. (H) The calculated correlation coefficients between the three kinetic parameters and the cell cycle at the time of infection (Correlation columns) or by taking cell division after infection into account (Partial correlation columns). (I) The initial CFP slopes are similar between cells that will divide and those that will not divide. (J) In cells that divide after CFP expression commenced, the slope significantly decreases after cell division. (K) Examples of trajectories from four cells that divided after CFP expression. The small red circles indicate the time of cell division. AU, arbitrary units.

The accumulation of CFP in infected cells followed a characteristic profile, which can be described by three parameters: (i) infection lag time (the time interval between viral adsorption and the expression of CFP), (ii) the rate of CFP accumulation (the slope of the line), and (iii) the total CFP accumulated during the time of imaging (the CFP concentration at the last time point). A schematic illustration of such a profile and the different parameters is shown in Fig. 5A. As seen in Fig. 5B to D, expression kinetics was highly variable among individual cells. The mean infection slope was 0.55 ± 0.4 (coefficient of variance [CV] = 0.74), the mean total CFP was 12.2 ± 10.7 (CV = 0.88), and the mean lag time was 5 ± 1.7 h (CV = 0.34).

While CFP kinetics in most cells followed this typical profile, in a portion of the cells (~10%), CFP expression began to slow down or stop completely at some point. We searched for a cellular explanation for this phenomenon and found that it is largely explained by cell division ensuing after the initiation of gene expression from the viral genome.

We found that during the time we monitored the cells (12 h after virus adsorption), 14% of the infected cells underwent cell division after the initiation of CFP expression. Two percent of the infected cells were in the early part of the cell cycle (0 to 12 h after mitosis) at the time of infection, and 12% were in the late part (12 to 24 h). We assessed the same kinetic parameters described above, separating the cells in each part of the cell cycle according to whether they divided after infection or not (Fig. 5E to G). Mitosis was found to correlate with slower CFP expression, as indicated by the decreased slope and total CFP in cells that underwent division following infection. This effect of cell division on kinetics is not dependent on the cell cycle stage of the cells, as the differences in the slope and total CFP were observed between cells that underwent mitosis and those that did not, rather than cells in different parts of the cell cycle (Fig. 5E and F). This finding is further supported by analysis of partial correlations (that is the correlation between two variables when taking into account the effect of a third variable) which shows that when cell division is taken into account, the correlation between the different kinetic parameters and the cell cycle stage at the time of infection diminishes or disappears (Fig. 5H).

Is there a direct impact of cell division on gene expression kinetics or is it the other way around and cells with slower expression kinetics are more likely to divide? To distinguish between these two possibilities, we performed two analyses. (i) We compared the initial CFP slope (the slope in the first hour after CFP expression) between cells that divided and those that did not. (ii) We compared the slope before and after cell division. We found that the initial CFP slopes are similar in both groups (Fig. 5I, *P* = 0.4), while the slopes after cell division were significantly lower than those before it (Fig. 5J, *P* = 2 × 10^−22^).

A detailed analysis of the dividing cells revealed that 70% of the cells that divided had a lower slope after division. The mean slope of these cells was 0.004 and was not significantly different from zero (*P* = 0.6), implying that no new CFP was being produced after cell division. A few examples of such CFP trajectories are shown in Fig. 5K.

Taken together, our findings show that cell division following gene expression from the viral genome occurs in 14% of infected cells and has a profound impact on expression kinetics.

### HSV-1 infection causes a sharp decline in SUMO2 and RPAP3 concentrations.

Having considered the effect of the cellular state on infection, we next turned to the secondary objective of our study—examining the effect of HSV-1 infection on the host cell. Of the 400 host proteins screened, we found 4 (1%) that significantly responded to infection.

**FIG 6:**
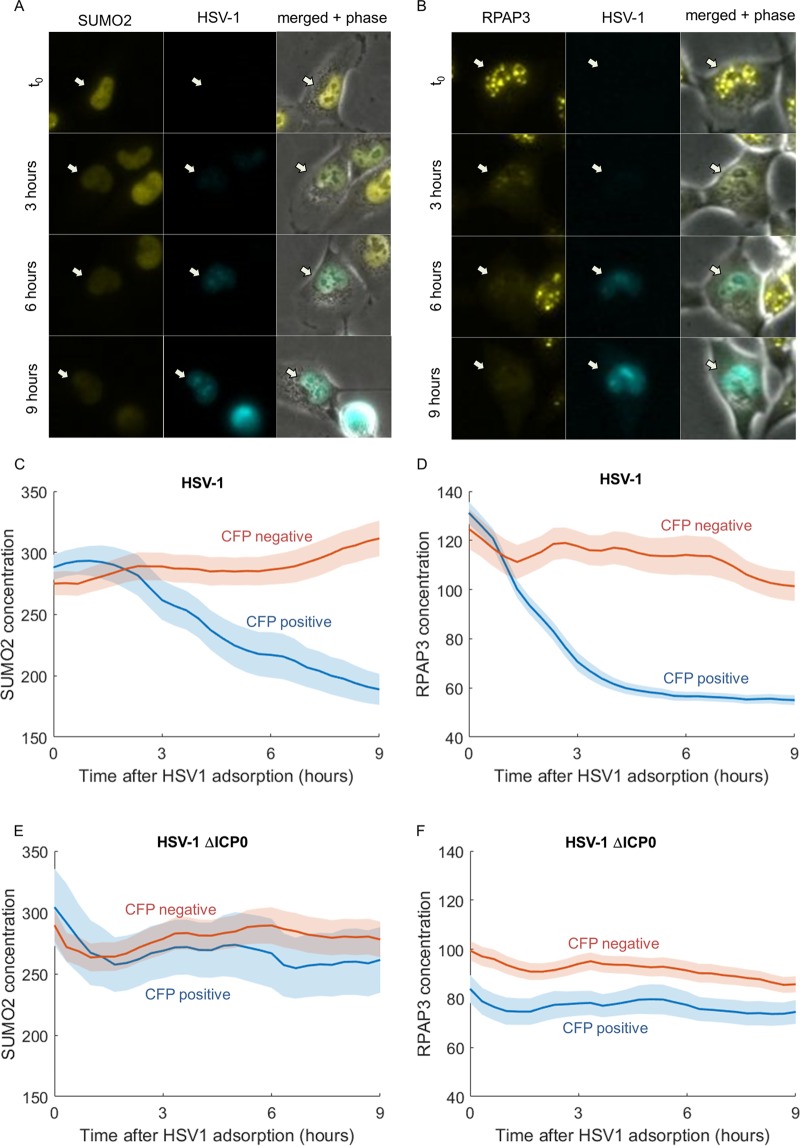
SUMO2 and RPAP3 levels are reduced upon HSV-1 infection. (A and B) Images of representative cells from time-lapse movies of YFP-tagged SUMO2 (SUMO2-YFP) (A) and RPAP3-YFP (B). (C and D) SUMO2 (C) and RPAP3 (D) concentrations in CFP-negative cells (red) and CFP-positive (blue) cells following HSV-1 adsorption. The colored lines show the means, and the colored areas show the SEMs. (E and F) SUMO2 (E) and RPAP3 (F) concentrations in CFP-negative (red) and CFP-positive (blue) cells following infection by a mutant HSV-1 that does not express ICP0. The colored lines show the means, and the colored areas show the SEMs.

The levels of two proteins, SUMO2 and RPAP3, were rapidly reduced in infected cells (Fig. 6). SUMO2 is a ubiquitin homolog that can be covalently attached to cellular proteins. Indeed, a decrease in SUMO2 levels upon HSV-1 infection has been previously reported to be mediated by the viral ubiquitin ligase ICP0 (47, 48). The SUMO2 concentration began dropping approximately 2 h after HSV-1 infection and continued to decrease up to 9 h after infection (Fig. 6A and C and Movie S1).

10.1128/mBio.01612-17.5 MOVIE S1  Time-lapse imaging of SUMO2-YFP following HSV-1 infection. Download MOVIE S1, AVI file, 2.3 MB. Copyright © 2017 Drayman et al. 2017 Drayman et al. This content is distributed under the terms of the Creative Commons Attribution 4.0 International license.

RPAP3 (RNA polymerase II-associated protein 3) has not been previously characterized in the context of HSV-1 infection. In noninfected cells, it showed distinct foci in the nucleus, which may represent transcriptional complexes on the cellular DNA (Fig. 6D). In HSV-1-infected cells, RPAP3 levels rapidly dropped, beginning at time zero and preceding CFP expression (Fig. 6B and D and Movie S2). The reduction in RPAP3 levels might relate to the previously observed changes in RNA polymerase II-dependent transcription following HSV-1 infection (49–53).

10.1128/mBio.01612-17.6 MOVIE S2  Time-lapse imaging of RPAP3-YFP following HSV-1 infection. Download MOVIE S2, AVI file, 2.8 MB. Copyright © 2017 Drayman et al. 2017 Drayman et al. This content is distributed under the terms of the Creative Commons Attribution 4.0 International license.

We tested whether the viral immediate early protein ICP0 is necessary for the degradation of these proteins (as previously reported for SUMO2) by infecting the cells with a mutant virus that does not express ICP0 and expresses a CFP reporter. Indeed, cells successfully infected by the ICP0 null mutant did not show reduced levels of either SUMO2 or RPAP3 (Fig. 6E and F). We validated these results using a nonfluorescent HSV-1 wild-type virus (KOS) and an independently derived ΔICP0 strain (7134 [54]). We infected the cells at an MOI of 3, fixed, and stained them with an anti-ICP4 antibody at 5 h postinfection. In cells infected with the wild-type virus, but not with the ΔICP0 mutant, a significant decrease in the levels of SUMO2 and RPAP3 was observed (Fig. S3).

10.1128/mBio.01612-17.3 FIG S3  KOS (wild type [wt]) infection, but not 7134 (DICP0) infection, causes a decrease in the levels of SUMO2-YFP and RPAP3-YFP. (A and C) Representative images of SUMO2-YFP (A) or RPAP3-YFP (C) clones at 5 h postinfection. Cells were infected with either HSV-1 strain KOS or 7134 at an MOI of 3, fixed, and stained with an antibody against ICP4. The merged image is pseudocolored for better visualization (YFP signal pseudocolored green and ICP4 signal pseudocolored red). An inset from each field is enlarged for better visualization. (B and D) Quantification of 40 cells from each condition and statistical analyses (one-tailed *t* test) of SUMO2-YFP (B) or RPAP3-YFP (D) infected cells. Only cells positive for ICP4 expression were analyzed. Download FIG S3, TIF file, 0.4 MB. Copyright © 2017 Drayman et al. 2017 Drayman et al. This content is distributed under the terms of the Creative Commons Attribution 4.0 International license.

### HSV-1 infection causes nuclear redistribution of SLTM and YTHDC1.

**FIG 7:**
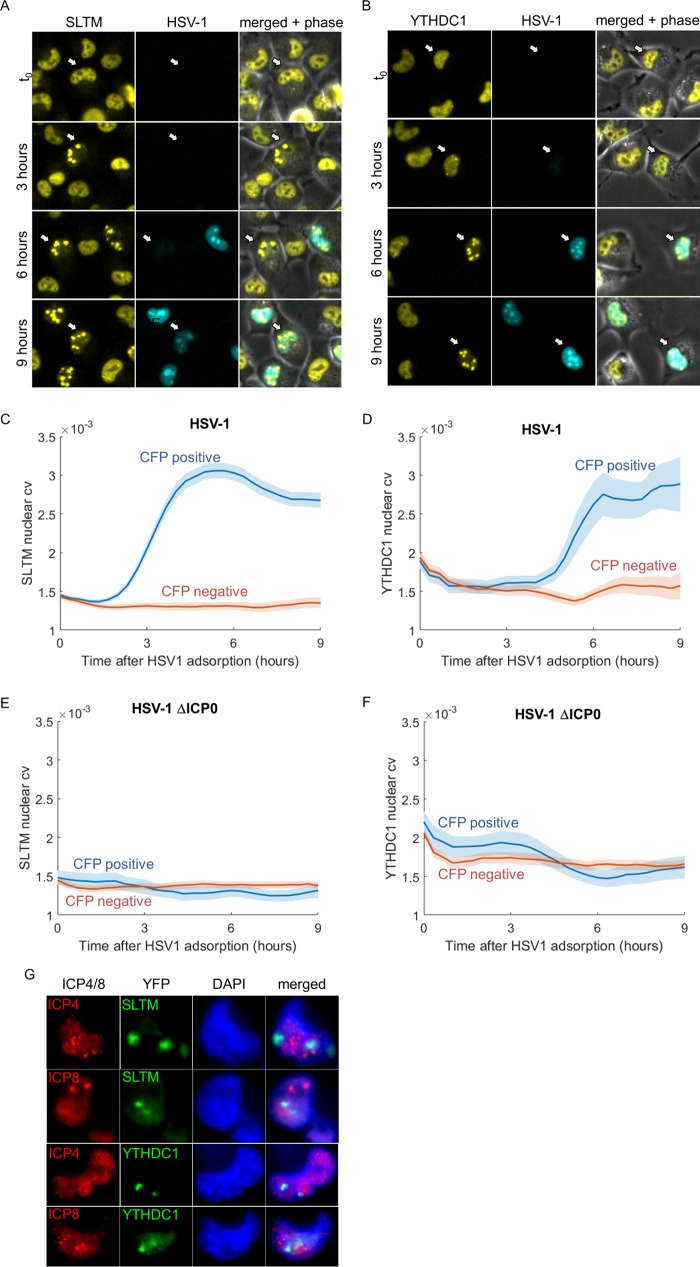
SLTM and YTHDC1 are redistributed upon HSV-1 infection. (A and B) Images of representative cells from time-lapse movies of SLTM-YFP (A) and YTHDC1-YFP (B). (C and D) SLTM (C) and YTHDC1 (D) nuclear coefficient of variance (cv) in CFP-negative (red) and CFP-positive (blue) cells following HSV-1 adsorption. The colored lines show the means, and the colored areas show the SEMs. (E and F) Nuclear cv of SLTM (E) and YTHDC1 (F) in CFP-negative (red) and CFP-positive (blue) cells following infection by a mutant HSV-1 that does not express ICP0. The colored lines show the means, and the colored areas show the SEMs. (G) Cells infected by HSV-1 were fixed and stained for ICP4 or ICP8 at 6 h after adsorption and imaged using an ×100 lens objective. Representative images of nuclear foci formed by SLTM (top two rows) or YTHDC1 (bottom two rows), which do not colocalize with ICP4 or ICP8 are shown. Cellular DNA was stained with 4′,6′-diamidino-2-phenylindole (DAPI) (blue).

Live-cell microscopy is able to observe changes in the localization of tagged proteins, which cannot be captured in conventional proteomic assays. We studied these changes by measuring the coefficient of variation (CV), which indicates how dispersed proteins are. We found that the CV of two nuclear proteins, SLTM and YTHDC1, increased specifically in CFP-positive cells, indicating a change in protein localization. The increase in the nuclear CV is a result of redistribution of these proteins upon infection, from being equally diffused around the nucleus to forming large foci (Fig. 7A to D and Movies S3 and S4).

10.1128/mBio.01612-17.7 MOVIE S3  Time-lapse imaging of SLTM-YFP following HSV-1 infection. Download MOVIE S3, AVI file, 4.1 MB. Copyright © 2017 Drayman et al. 2017 Drayman et al. This content is distributed under the terms of the Creative Commons Attribution 4.0 International license.

10.1128/mBio.01612-17.8 MOVIE S4  Time-lapse imaging of YTHDC1-YFP following HSV-1 infection. Download MOVIE S4, AVI file, 2.5 MB. Copyright © 2017 Drayman et al. 2017 Drayman et al. This content is distributed under the terms of the Creative Commons Attribution 4.0 International license.

The redistribution of YTHDC1 and SLTM could be a result of their recruitment to viral replication compartments. To test this, we fixed infected cells 6 h after infection and stained them for either ICP4 (an immediate early protein that is required for HSV-1 gene expression) or ICP8 (an early protein required for HSV-1 genomic replication) and found that the nuclear foci of SLTM and YTHDC1 do not colocalize with replication compartments (Fig. 7G). In fact, in agreement with the rapid kinetics of the appearance of these foci, we occasionally observed cells that contained such foci but were negative for ICP8, suggesting that the redistribution happens before viral DNA replication and is mediated by one of the immediate early proteins of the virus.

We tested whether ICP0 is also involved in the redistribution of these proteins and found that cells successfully infected with the mutant virus did not show redistribution of both proteins (Fig. 7E and F). Similar results were obtained with the wild-type KOS strain and the ΔICP0 7134 strain (Fig. S4).

10.1128/mBio.01612-17.4 FIG S4  KOS (wt) infection, but not 7134 (DICP0) infection, causes a redistribution of YTHDC1-YFP and SLTM-YFP. (A and C) Representative images of YTHDC1-YFP (A) or SLTM-YFP (C) clones at 5 h postinfection. Cells were infected with either HSV-1 strain KOS or 7134 at an MOI of 3, fixed, and stained with an antibody against ICP4. The merged image is pseudocolored for better visualization (YFP signal pseudocolored green and ICP4 signal pseudocolored red). An inset from each field is enlarged for better visualization. (B and D) Quantification of 40 cells from each condition and statistical analyses (one-tailed *t* test) of YTHDC1-YFP (B) or SLTM-YFP (D) infected cells. Only cells positive for ICP4 expression were analyzed. Download FIG S4, TIF file, 0.5 MB. Copyright © 2017 Drayman et al. 2017 Drayman et al. This content is distributed under the terms of the Creative Commons Attribution 4.0 International license.

## DISCUSSION

We present here the first application of the dynamic proteomic approach to study the impact of cell-to-cell variability on viral infection. We find that the ability to initiate gene expression from the viral genome is modulated by the cellular state at the time of infection. The cell’s cell cycle, velocity, and local cell density are predictive of viral gene expression success, suggesting that individual cells have different capacities to fight off or support HSV-1 infection.

We find that cell velocity and local cell density correlate with the probability of successful gene expression initiation. Cell movement requires the generation of membrane extensions called filopodia (55), which HSV-1 has been reported to “surf” as a mechanism of entry into the host cell (reviewed in reference 56). Thus, increased cell movement might be linked to a higher chance of a virus to catch a filopodium and enter the cell. A similar relation was shown between the local cell density and the cellular endocytic machinery (18). An alternative explanation might be that motile cells sample more of their environment during the 30 min of virus adsorption, thus increasing the chances of virus-cell encounters taking place.

We find that the cell cycle position influences the probability to initiate gene expression from the viral genome, exemplified by the large effect size of RFX7 and geminin concentration. This cell cycle effect also explains the small effect size (<20%) observed for most YFP-tagged host proteins in our screen (Fig. 2A), since the concentrations of most host proteins showed a low correlation with the cell cycle (not shown). The dynamics of geminin and RFX7 in cells following infection seem to be different (Fig. 2C and D), probably representing a random difference in the position of these cells along the cell cycle. This is supported by the fact that in validation experiments the difference between the CFP^+^ and CFP^−^ cells at time zero remained reproducible, while the dynamics at later times varied (data not shown).

The influence of the cell cycle on biological systems is often ignored or considered an artifact of *in vitro* experiments. However, if this was indeed the case, one should wonder why the virus evolved such elaborate mechanisms to arrest the cell cycle? (The interactions between herpesviruses and cell cycle are reviewed in reference 57.) HSV-1 infection is known to interfere with the natural progress of the cell cycle, with infection usually resulting in the inhibition of cellular DNA synthesis and cell cycle arrest at the G_1_/S checkpoint (23–26). In addition, infection of cells already in the G_2_ phase results in inhibition of cell division and arrests the cells at the G_2_/M checkpoint (26). The major protein implicated in the dysregulation of the cell cycle is the immediate early protein ICP0 (26–28), although other viral proteins have also been implicated (29, 30). It was also recently shown that infected cells can manipulate the cell cycle in their neighboring (uninfected) cells by driving them to initiate DNA synthesis through a secreted agent (58).

Our results appear to be in contrast to two previous works that, using population-level analyses, concluded that the cell cycle stage at the time of infection does not influence virus propagation (59, 60). However, as discussed below, the results of these papers do not seem to be in conflict with the ones presented here.

Cohen et al. (59) measured the amount of viral progeny produced by KB cells infected at a high MOI of 200 at various times after cell cycle synchronization. They collected intracellular viruses, assayed the number of viruses by plaque assay (on asynchronous cells), and found that the number of viruses produced was the same, irrespective of the cell cycle stage of the cells. We found that when cells are infected at a low MOI of 0.5, they are capable of initiating gene expression throughout the cell cycle, but they are less likely to do so as they approach mitosis. Thus, our results suggest that given a high enough MOI, all cells are expected to initiate viral gene expression and successfully finish the viral life cycle, as observed by Cohen et al. (59).

Bringhurst and Schaffer (60) have studied the effect of the cell cycle on viral progeny production by synchronizing Vero cells to G0/G_1_ using four combinations of nutrient deprivation conditions and performing plaque assays on the synchronized monolayers. The authors concluded that the cell cycle stage at the time of infection did not significantly affect plaque formation. However, their results show that in three of the four synchronization protocols, a slight decrease (ca. twofold) is observed as the cells progress through the cell cycle (see Fig. 1D to J in reference 60). This effect is similar in magnitude to the effect we have measured here, ranging between 1.5- and 2.7-fold difference (Fig. 3D and E). We assume that the authors regarded this decrease as nonsignificant due to the fact that the plaque assay, while being the gold standard to measure the production of viable progeny, is not sensitive enough to measure small effects. Our single-cell analysis offers a much improved sensitivity and can significantly determine such effects, but unfortunately, it can only monitor gene expression and not production of viable progeny. Thus, the results of Bringhurst and Schaffer do not stand in contrast to ours and may in fact support them. This apparent disagreement serves to highlight the need for the development of more-sensitive, single-cell-level assays for the production of viable progeny.

We further find that cell division can arrest gene expression from the viral genome after it has been initiated. Of the cells that divided after infection, 70% showed no increase in CFP following mitosis. This suggests that during mitosis something happens to inhibit further expression from the viral genome. One possibility is that during mitosis the virus genome becomes silenced and cannot resume transcription. Another possibility is that since cell division involves the breakdown of the nuclear envelope, after division the viral genome ends up in the new cell cytoplasm, where it lacks the appropriate machinery for transcription. This intriguing finding should be further explored to decipher the molecular mechanisms underlying it. It is important to remember our model system here is human cancer cells, which might be more inclined to divide than untransformed cells. In this context, we should note that HSV-1 lytic infection does occur in dividing cells in the epithelia, such as keratinocytes. It could also prove important in the major efforts of using HSV-1-based vectors as oncolytic viruses for the treatment of cancer.

The finding that gene expression kinetics vary so widely among individual cells is interesting, since expression kinetics can have a dramatic effect *in vivo*, where viral replication and dissemination are in a race against the host’s mounting antiviral response. In this context, identifying the cellular determinants of gene expression kinetics could prove valuable for our basic understanding of infection biology.

Looking at the individual proteins in our screen, we find two proteins whose concentrations at time zero are indicative of successful gene expression, and both are markers of the cell cycle. However, this should not be interpreted to suggest that other cellular proteins not related to the cell cycle do not influence infection outcome. Specifically, due to the way in which it was constructed (39), our library does not include known antiviral effectors, such as proteins in the NF-κB and interferon pathways, as these proteins have low expression under basal conditions. Future studies looking into the effect of heterogeneity in such proteins on viral infection are likely to better shape our understanding of the interplay between host and virus.

We identify four proteins that are modulated by infection. These four proteins represent 1% of the proteins studied, a similar fraction as was recently found by a conventional proteomic study on the population level (36). Three of these proteins (RPAP3, YTHDC1, and SLTM) have not been previously described in the context of HSV-1 infection. Interestingly, all three of these proteins seem to be related to the life cycle of mRNA.

RPAP3 (RNA polymerase II-associated protein 3) is one of the four components of the R2TP complex (61), a complex responsible for the assembly of several cellular machineries, including the RNA polymerase II complex. The rapid reduction in RPAP3 levels is in line with the previous reported alterations in RNA polymerase II upon HSV-1 infection (50–53) and the known function of the virus virion host shutoff protein (vhs) that arrests the host’s translation by degrading mRNA. The rapid block of cellular transcription and translation is likely to be important for hampering the innate immune response in the infected cells, as the vhs protein has been shown to attenuate the host’s antiviral response (62, 63).

SLTM (SAF-B-like transcription modulator) is a scarcely studied homolog of SAF-B (scaffold attachment factor B). Overexpression of SLTM in HeLa cells results in transcriptional repression and cell death (64). SAF-B is involved in the spatial arrangement of chromatin loops, poising them for transcription (65). It can also directly bind to RNA and was shown to participate in the alternative splicing of different genes (66).

YTHDC1 is also a regulator of alternative splicing, which specifically recognizes and binds N^6^-methyladenosine (m^6^A)-containing RNAs (67). YTHDC1 was shown to physically interact with SAF-B (65), and all three proteins (YTHDC1, SLTM, and SAF-B) were found to bind to the Xist long noncoding RNA that is required for X-chromosome inactivation (68). We find that upon HSV-1 infection, SLTM and YTHDC1 change localization, aggregating in large nuclear foci. A similar observation was made for SAF-B upon heat shock treatment (69). However, in the context of HSV-1 infection, the redistribution of YTHDC1 and SLTM to nuclear foci seems to be actively controlled by the virus, as it requires the expression of the immediate early protein ICP0.

One possible role for the sequestration of these proteins by HSV-1 could be the repression of gene splicing. Unlike the majority of viruses that replicate in the nucleus, HSV-1 genome contains very few introns (only 5 genes out of the ~80 genes carried on the virus) and thus requires very little splicing activity. Indeed, several works have indicated that HSV-1 actively represses splicing in the host (70–72). This however has been called into question by a recent study that found that HSV-1 causes widespread disruption of host transcription termination and that ensuing read-in into neighboring genes is responsible for the apparent inhibition of splicing (73). In this regard, it is interesting to note that m^6^A modification of mRNA (which is recognized by YTHDC1) is most abundant near transcriptional termination sites (74, 75). Whether the redistribution of YTHDC1 and SLTM is linked to changes in the host alternative splicing or aberrant transcription termination is an intriguing question that requires further exploration.

In conclusion, we report here on the first direct experimental evidence that single cells differ in their capacity to initiate gene expression from the HSV-1 genome. While the outcome of a virus-host encounter is far from being deterministic, it is also not completely stochastic, and different cellular features modulate the chance of the virus to establish a successful infection. Dynamic proteomics and other time-resolved measurements can now be used to study this for other viral infections, and ultimately to search for common cellular and molecular features that modulate infection at the single-cell level.

## MATERIALS AND METHODS

### Library of annotated clones.

The generation of the library of annotated clones (LARC) was described elsewhere (39). The library consists of more than 1,000 clones of the non-small cell lung carcinoma cell line H1299. All clones share the constitutively expressed mCherry-tagged proteins. The mCherry signal is bright in the nucleus and dim in the cytoplasm and is used for the automated segmentation and tracking of the cells during analysis of time-lapse movies. Each of the clones in the library also expresses a unique yellow fluorescent protein (YFP)-tagged, full-length protein. Tagging was done by the central dogma (CD) tagging scheme, such that one copy of the gene is tagged in its native chromosomal location, and is thus under the control of its endogenous promoter. For all the clones in the library, the YFP-tagged protein shows a correct subcellular localization (clones that did not show correct localization were discarded). Cells were grown in RPMI 1640 supplemented with penicillin, streptomycin, and 10% fetal bovine serum at 37°C and 8% CO_2_. Cells were regularly tested for mycoplasma.

### Viruses.

Herpes simplex virus 1 (HSV-1) strain 17 was constructed to express mTurq2 (a brighter derivative of cyan fluorescent protein [CFP]) from the cytomegalovirus (CMV) immediate early promoter inserted between UL37 and UL38 genes in the viral genome. The CMV promoter was derived from Clontech’s pEGFP-N1 (EGFP stands for enhanced green fluorescent protein) plasmid (for sequence, see https://www.addgene.org/vector-database/2491/). The ICP0 mutant was constructed similarly to express mTurq2 based on the HSV-1 dl1403 (76) as previously described (77). KOS and 7134 (54) strains were a kind gift from Matthew Weitzman.

### HSV-1 titration and infection.

CFP-expressing HSV-1 was titrated in Vero cells using the plaque assay. We assessed the infectivity of HSV-1 on H1299 cells by determining the percentage of CFP-positive cells 9 h after adsorption when the cells were infected with serial dilutions starting at a multiplicity of infection (MOI) of 10. We compared the observed percentage of CFP-positive cells to that expected from the MOI and found that H1299 cells are approximately 10-fold less susceptible to infection than Vero cells. We used this to determine the MOI for all experiments. Thus, an MOI of 0.5 when infecting H1299 cells is equivalent to an MOI of 5 when infecting Vero cells.

### Cell plating, infection, and imaging.

Cells were plated and imaged in 12-well, glass-bottom plates (MatTek, MA, US). One hour before the cells were plated, each well on the plate was coated with 200 µl of 10 µg/ml fibronectin from bovine serum (Sigma, Israel). Each well on a plate was washed once with 1 ml phosphate-buffered saline (PBS), and 10^5^ cells were plated in each well. The cells were allowed to grow for 24 h. The following day, the medium was replaced by an imaging medium (transparent RPMI 1640 without phenol red and riboflavin from Biological Industries, Israel, supplemented with penicillin, streptomycin, and 5% fetal bovine serum) approximately 1 h before infection. The medium was then aspirated, and 300 µl of imaging medium containing HSV-1 at an MOI of 0.5 was added. Virus was allowed to adsorb to the cells for 30 min at 37°C. During this time, the imaging setup was performed—calibrating the microscopes, choosing four fields of view for each well, and setting the acquisition times for the fluorescent channels. After 30 min, the virus-containing medium was aspirated, and 2 ml of imaging medium was added to each well. The plates were placed in a temperature-, CO_2_-, and humidity-controlled chamber in the microscope, the focus was adjusted, and imaging started. Imaging was performed using two inverted epifluorescence Leica microscopes (DMIRE2 and DMI6000b), controlled by macro scripts developed in-house.

### Image and data analysis.

Flat field correction and background subtraction were performed for all images prior to starting the analysis. Cell segmentation and tracking were done using the PhenoTrack package for Matlab, previously developed in our lab (78), with some additions and modifications. All codes used in this work are available upon request. The package was extended to extract morphological and textural features. The CFP concentration was calculated as the total CFP fluorescent area divided by the cell area. To ensure correct tracking of the cells, we employed several filters, eliminating trajectories of cells that did not meet certain criteria. Such criteria included, for example, more than one mitosis event in 12 h and a rapid, nonphysiological, change in the mCherry levels. Overall, a third of data passed the various quality control filters, resulting in ~52,000 reliably tracked cells out of ~190,000 cells imaged in total.

### Cell cycle synchronization.

A total of 5 × 10^4^ cells were plated in 12-well glass bottom plates as described above. At 5 p.m. the medium was replaced with a growth medium containing 2 mM thymidine (Sigma-Aldrich, Israel). At 8 a.m. the next morning, cells were washed twice with PBS, and growth medium was added. At 5 p.m. of the same day, the medium was again replaced with medium containing thymidine. At 8 a.m. the next morning, half of the wells were released from blocking (washed twice and given new growth medium), and half were maintained in the blocking medium. At 4 p.m., 8 h later, the blocked cells were released. The cells were washed and infected with HSV-1 at an MOI of 0.25 or 0.5 and imaged as described above. One well from each condition (15 min or 8 h postrelease) was harvested and fixed with 70% ethanol at the time of HSV-1 infection. Samples were rehydrated, treated with RNase A, and stained with propidium iodide to analyze the cell cycle stage by fluorescence-activated cell sorting (FACS).

### Supervised machine learning for predicting infection outcome.

We divided our data set into independent training and test sets. To avoid any biases due to differences between the clones, we made sure that each clone is similarly represented in the infected and noninfected groups. We additionally made sure that no particular clone would be overrepresented in the data set. Eighty percent of the data were used to train the classifier, and 20% (from clones not used in the training step) were used to test its performance. Machine learning was done using Matlab’s *fitglm* function.

### Extracting cell cycle data from still images.

We employed a supervised machine learning approach, similar to that used by others (43–45), which infers the position of a cell in the cell cycle from a still image using a random forest regression predictor. The performance of this predictor is shown in Fig. 3. We trained and tested the predictor using independent data sets of cells that divided during the movies, so that we could determine the time after mitosis for each cell in each frame. We aligned the cell trajectories to an imaginary cell cycle length of 24 h. This gave the best results, but using other cell cycle lengths did not significantly alter our findings.
