# Supplementary figures and images for: Dynamic Proteomics of Herpes Simplex Virus Infection
Source: mBio. 2017 Nov 7;8(6):e01612-17. doi: 10.1128/mBio.01612-17 (PMC5676043; doi:10.1128/mBio.01612-17)

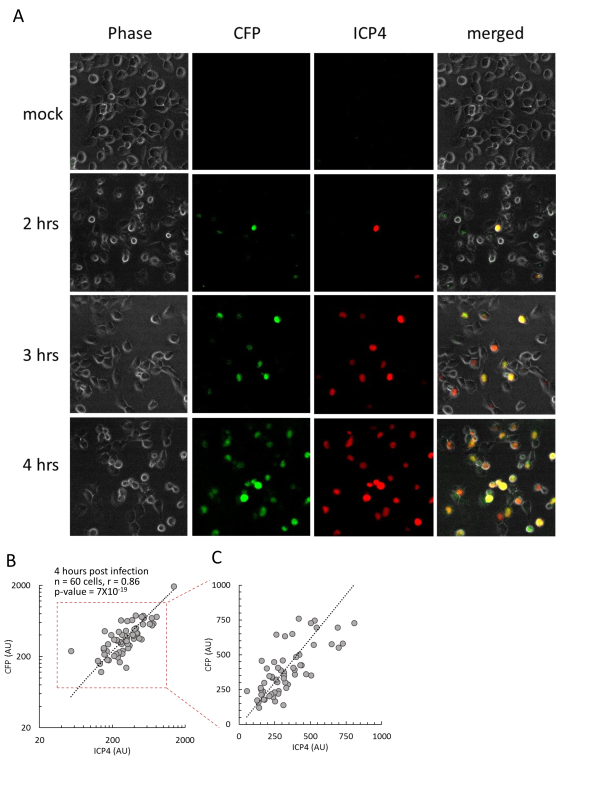

Supplement: FIG S1 [file mbo006173578sf1.tif]

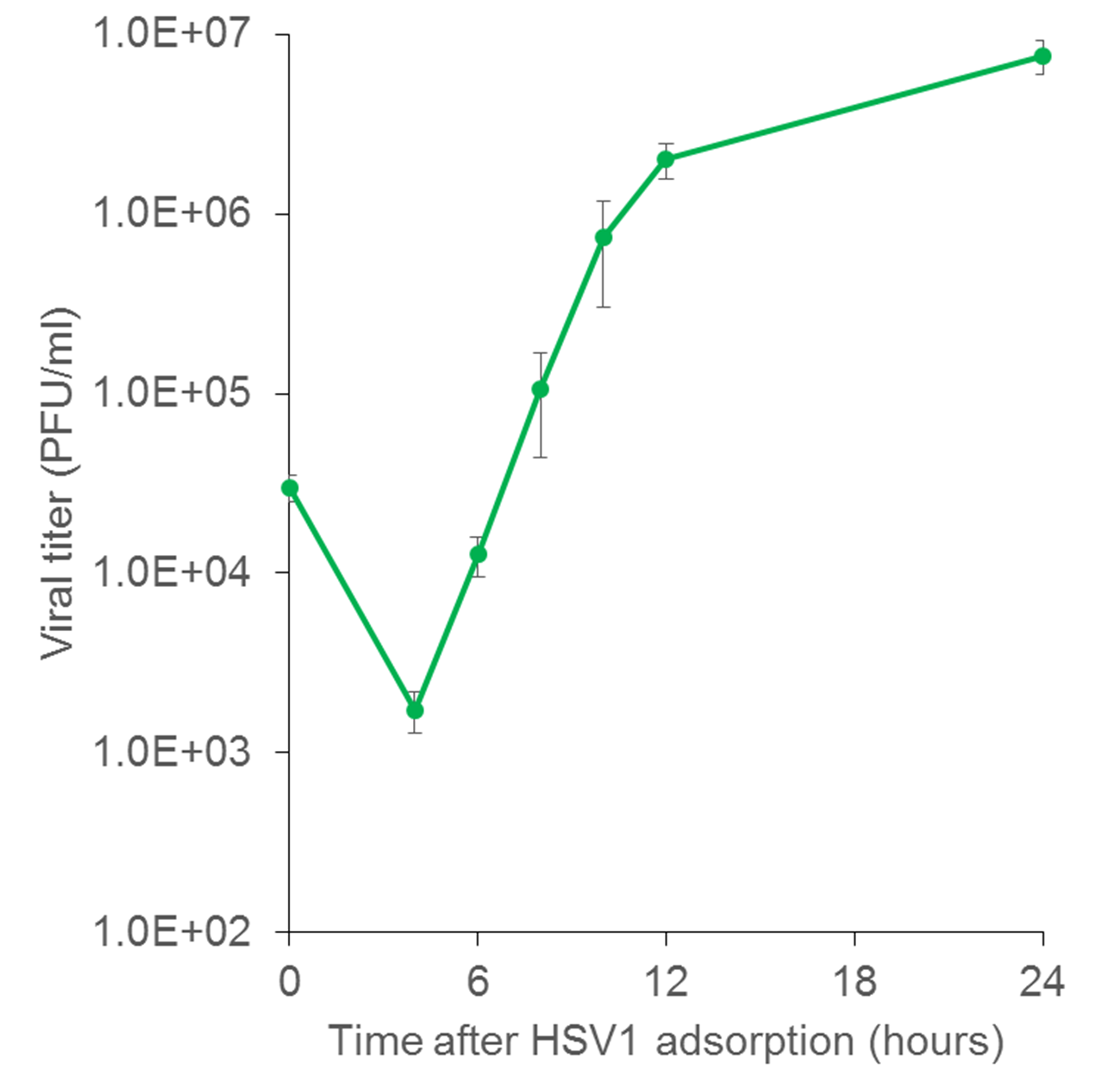

Supplement: FIG S2 [file mbo006173578sf2.tif]

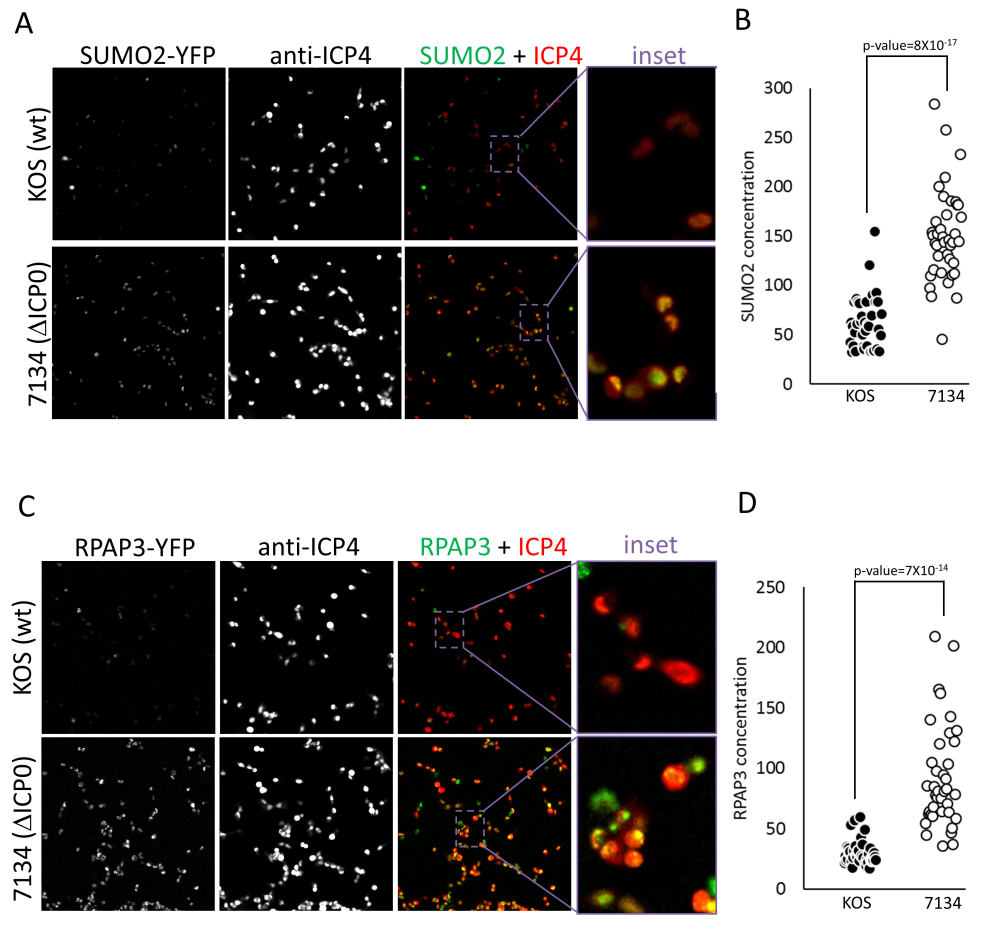

Supplement: FIG S3 [file mbo006173578sf3.tif]

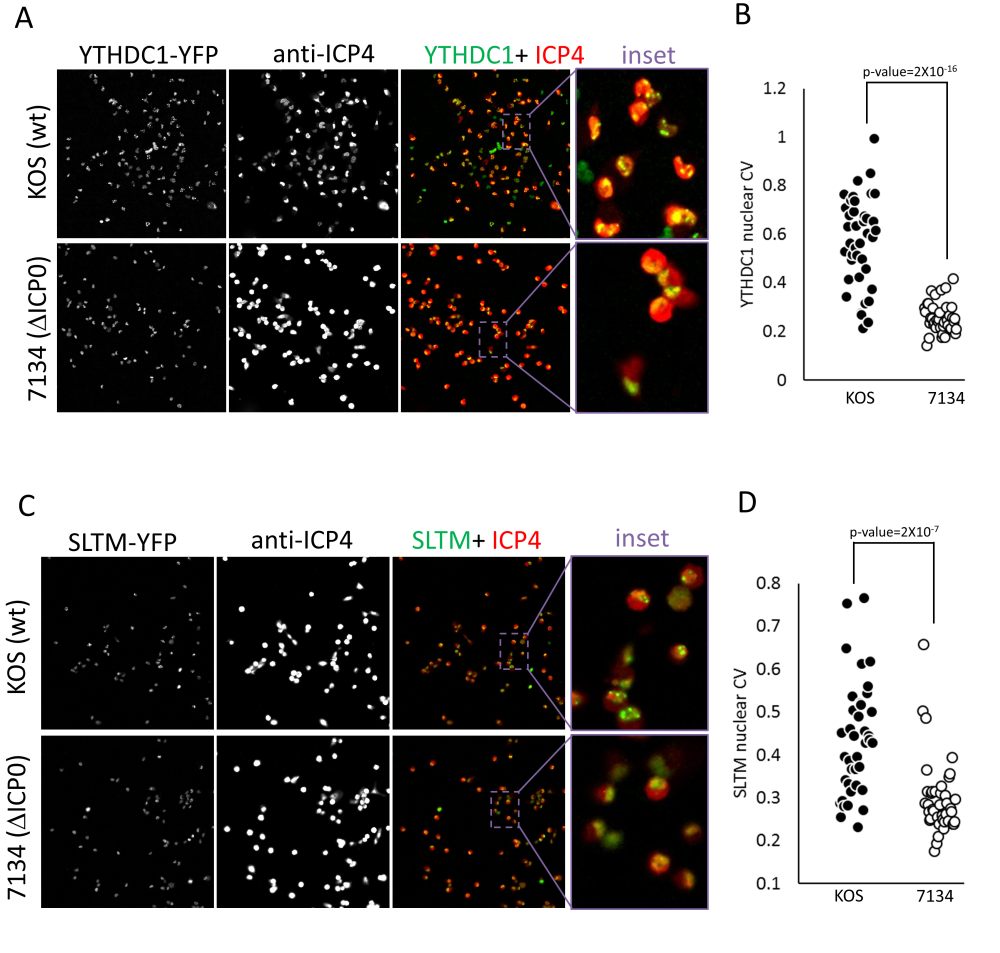

Supplement: FIG S4 [file mbo006173578sf4.tif]
